# Microglia Activate Migration of Glioma Cells through a Pyk2 Intracellular Pathway

**DOI:** 10.1371/journal.pone.0131059

**Published:** 2015-06-22

**Authors:** Kimberleve Rolón-Reyes, Yuriy V. Kucheryavykh, Luis A. Cubano, Mikhail Inyushin, Serguei N. Skatchkov, Misty J. Eaton, Jeffrey K. Harrison, Lilia Y. Kucheryavykh

**Affiliations:** 1 Department of Biochemistry, Universidad Central del Caribe, School of Medicine, Bayamón, Puerto Rico, United States of America; 2 Department of Anatomy and Cell Biology, Universidad Central del Caribe, School of Medicine, Bayamón, Puerto Rico, United States of America; 3 Department of Physiology, Universidad Central del Caribe, School of Medicine, Bayamón, Puerto Rico, United States of America; 4 Department of Pharmacology and Therapeutics, College of Medicine, University of Florida, Gainesville, Florida; University of Michigan School of Medicine, UNITED STATES

## Abstract

Glioblastoma is one of the most aggressive and fatal brain cancers due to the highly invasive nature of glioma cells. Microglia infiltrate most glioma tumors and, therefore, make up an important component of the glioma microenvironment. In the tumor environment, microglia release factors that lead to the degradation of the extracellular matrix and stimulate signaling pathways to promote glioma cell invasion. In the present study, we demonstrated that microglia can promote glioma migration through a mechanism independent of extracellular matrix degradation. Using western blot analysis, we found upregulation of proline rich tyrosine kinase 2 (Pyk2) protein phosphorylated at Tyr579/580 in glioma cells treated with microglia conditioned medium. This upregulation occurred in rodent C6 and GL261 as well as in human glioma cell lines with varying levels of invasiveness (U-87MG, A172, and HS683). siRNA knock-down of Pyk2 protein and pharmacological blockade by the Pyk2/focal-adhesion kinase (FAK) inhibitor PF-562,271 reversed the stimulatory effect of microglia on glioma migration in all cell lines. A lower concentration of PF-562,271 that selectively inhibits FAK, but not Pyk2, did not have any effect on glioma cell migration. Moreover, with the use of the CD11b-HSVTK microglia ablation mouse model we demonstrated that elimination of microglia in the implanted tumors (GL261 glioma cells were used for brain implantation) by the local in-tumor administration of Ganciclovir, significantly reduced the phosphorylation of Pyk2 at Tyr579/580 in implanted tumor cells. Taken together, these data indicate that microglial cells activate glioma cell migration/dispersal through the pro-migratory Pyk2 signaling pathway in glioma cells.

## Introduction

Glioblastoma (GBM) is an extraordinarily aggressive type of brain cancer due to resistance to radiation and chemotherapy and the highly invasive nature of this tumor. A single GBM cell can invade throughout the brain and often produce secondary lesions at sites distant from the primary tumor [1], thus, reducing the efficacy of surgical resection [2, 3]. The tumor microenvironment has a critical role in tumor invasion and progression with microglia as a significant player. The amount of microglial infiltration of the tumor is associated with poor clinical prognosis in patients with high graded gliomas [4, 5, 6]. Accumulating evidence demonstrates a role for microglia in tumor growth [7, 8, 9, 10, 11, 12], but the molecular mechanisms through which tumor cells interact with their environment to regulate migration from primary tumor sites are not well investigated.

Microglial cells comprise up to 30% of GBM total tumor mass [13, 14], and therefore constitute a potentially important component of the microenvironment of these tumors. Microglial cells in gliomas undergo a morphological transformation and are capable of some innate immune responses such as phagocytosis and cytotoxicity. Paradoxically, glioma infiltrating microglia do not secrete some key cytokines such as IL-6, IL-1β and TNF-α [1, 15] that are critical to develop effective immune responses. In fact, it has been shown that tumor infiltrating microglia increase the infiltrative behavior of glioma cells increasing proteinase activity and degradation of the extracellular matrix in the tumor area [4, 5, 7, 8] as well as stimulate glioma cell proliferation and dispersal into surrounding healthy brain areas [5, 16, 17, 18]. Membrane type 1 metalloprotease (MT1-MMP), matrix metalloproteinase-2 (MMP2), cathepsin B, and urokinase receptor (uPAR) are overexpressed in gliomas and they are postulated to play central roles in breaking down the extracellular matrix in the tumor area and, thereby, creating pathways for tumor cells invasion [7, 8, 19, 20].

Proline-rich tyrosine kinase (Pyk2) is a member of the focal adhesion kinase (FAK) family. Pyk2 integrates signals from cell adhesion, growth factor, and G-protein-coupled receptors and has a key role in migration of specific cell types, particularly, in leukocytes and fibroblasts [21, 22]. Pyk2 plays an important role in cell motility and invasion [21, 22, 23, 24, 25], and Pyk2 expression is shown to occur frequently in human astrocytomas with a significant correlation between the grade of malignancy of astrocytomas and the expression of Pyk2 [26]. Inhibition of Pyk2 activity in glioma cells significantly reduced tumor invasion and increased survival in mice with glioma cell xenografts [27].

Involvement of microglia in Pyk2 signaling in glioma cells has never been published although Pyk2 has been identified as an important regulator of glioma cell migration. In this report, we have identified Pyk2 as a new intracellular signaling element mediating interactions between microglia and glioma cells which lead to activation of glioma cell migration. We hypothesize that microglia can stimulate glioma cell dispersal not just through degradation of the extracellular matrix, but also by directly activating intracellular signaling pathways in glioma cells.

To analyze the activation of Pyk2 in glioma cells in response to soluble factors released by microglia we investigated GL261 murine glioma cells, C6 rat glioma cells, and three different human glioma cell lines with varying levels of invasiveness. A172 glioblastoma cells are non-tumorigenic in anti-thymocyte serum treated NIH Swiss mice. U-87MG cells are tumorigenic and highly invasive glioblastoma, whereas HS683 are non-invasive non-tumorigenic low graded glioma [28, 29, 30]. Many signaling pathways may be affected in different types of glioma in response to soluble factors released by microglia. Using different glioma cell lines, we have identified a common and major signaling pathway that is exploited to increase glioma migration and invasiveness.

## Materials and Methodology

### Ethics Statement

All procedures involving rodents were conducted in accordance with the National Institutes of Health regulations concerning the use and care of experimental animals. All procedures involving animals were approved by Universidad Central del Caribe Institutional Animal Care and Use Committee. All efforts were made to minimize suffering.

### Cell Culture

A172, U87, HS683 human glioma cell lines and C6 rat glioma cell line were obtained from ATCC (Manassas, VA). The GL261 glioma cell line derived from C57BL/6 mice was obtained from the NCI (Frederick, MD). Previously established CHME5 immortalized human fetal microglia [31] were used for experiments with human glioma cell lines. C57BL/6 mouse and Sprague Dawley rat primary microglial cultures were prepared in our lab and used for experiments with GL261 and C6 cells, respectively. All cells were cultured in Dulbecco’s modified Eagle medium (DMEM) supplemented with 10% fetal calf serum, 0.2 mM glutamine and antibiotics (50 U/mL penicillin, 50 μG/ml streptomycin) and maintained in a humidified atmosphere of C0_2_/air (5%/95%) at 37°C. The medium was exchanged with fresh culture medium about every 2–3 days.

### Primary microglia cultures

Mouse and rat primary microglia cultures were obtained from cortical mixed glial cultures of 1–3 day old C57BL/6 mice and Sprague Dawley rats respectively as described by Markovic et al. 2005 [7]. Briefly, mixed glial cultures were prepared from neocortex of 1–3 day old rodents. Brains were removed after decapitation and the meninges stripped away to minimize fibroblast contamination. The forebrain cortices were dissociated using the stomacher blender method. The cell suspension was then allowed to filter by gravity through a #60 sieve and then through a #100 sieve. After centrifugation, the cells were suspended in DMEM (containing 30 mM glucose, 2 mM glutamine, 1 mM pyruvate and 10% fetal bovine serum, 100 units/ml of penicillin/streptomycin) and plated in 75 cm^2^ flasks at a density of 300,000 cells/cm^2^. The medium was exchanged with fresh culture medium about every 5 days. At confluence (about 12–14 days), microglial cells were collected by a mild shaking (1 h at 125 rpm). The medium was transferred to a new flask and incubated for 30 min before removal of non-adherent cells by changing the medium. The purity of the microglial culture was assessed by immunocytochemical detection of isolectin B4, a microglial marker [32].

### Preparation of Conditioned Medium

For glioma/microglia co-culturing glioma and microglial cells were collected by treatment with trypsin 2X and seeded (1X10^5^) in 60mm dishes in a ratio 2:1. Co-cultured cells were incubated in 2mL of DMEM with 10% FBS for 24 hours prior to obtaining activated microglia conditioned medium (AMCM) for the experiments. In addition, medium, conditioned from microglia (MCM) alone was used as well as medium conditioned from glioma cells pretreated with AMCM—glioma conditioned medium (GCM). Conditioned mediums were centrifuged to remove debris and dead cells and used for the experiments immediately.

### Invasion/Migration Assays

Invasion assays were performed using modified Boyden chambers [33] coated with Matrigel. 8 μM pore transwells (Falcon HTS FluoroBlock Inserts, BD Biosciences, Bedford, MA, cat. # 351152) were pre-coated with 30 μL of BD Matrigel^TM^ Matrix (BD Biosciences, Bedford, MA, cat. # 354263) for 2 hours at 37°C. Glioma cells (50,000 cells) were placed on top of the membrane in the upper chamber in 150 μL of DMEM and microglia (50,000 cells) were placed in the lower chamber in 500 μL of DMEM. The control group did not contain microglia. Invasion was allowed to proceed for 18 hours at 37°C, 5% CO_2_. In migration assays, Matrigel that mimics the extracellular matrix was not used. Migration assays were performed for 5 hours at 37°C, 5% CO_2_.

In both assays, migratory cells were fixed with 100% methanol and stained with 40 μg/μL Propidium iodide (PI). Images of the cells that migrated to the lower chamber were captured using an inverted microscope Olympus BX51WI (Olympus, Shinjuku, Tokyo, Japan), Qcolor 3 camera (Olympus) and Q-capture Pro software (Olympus) and the number of migrating and invading cells on the underside of the filter were counted. The mean of the total invading or migrating cells was determined from 3 independent experiments.

### Cell Signaling Cascade Antibody Microarrays

Panorama Antibody Microarray—Cell Signaling Kits to total and phosphoproteins (Sigma Chemical Co., St. Louis, MO, cat. # CSAA1) were utilized and processed according to manufacturer’s instructions. Proteins used for array hybridization were extracted and labeled with biotin using the Full Moon Biosystems, Inc. (Sunnyvale, CA) Antibody Array Assay Kit (Cat. # KAS02) as per the manufacturer’s instructions. Antibody Microarrays were sent to Full Moon Biosystems, Inc. for measurements of the fluorescent signals.

### Intracranial Implantation of Glioma Cells

All surgery was performed under isoflurane anesthesia, and all efforts were made to minimize suffering. GL261 glioma cells were implanted into the right cerebral hemisphere of 12–16 week old C57BL/6 mice. Implantation was performed according the protocol that we described earlier [34, 35]. Briefly, mice were anesthetized with isoflurane and a midline incision was made on the scalp. At stereotaxic coordinates of bregma, 2mm lateral, 1mm caudal and 3mm ventral a small burr hole (0.5mm diameter) was drilled on the skull. 1 μL of cell suspension (2X10^4^ cells/μL in PBS) was delivered at a depth of 3 mm over 2 min. Sixteen days following injection, animals were anesthetized with pentobarbital (50 mg/kg) and transcardially perfused with PBS followed by 4% paraformaldehyde (PFA). Brains were removed and postfixed in 4% PFA/PBS for 24 h at 4°C, followed by 0.15M, 0.5M, and 0.8M sucrose at 4°C until fully dehydrated. Brains were then frozen-embedded in Cryo-M-Bed embedding compound (Bright Instrument, Huntingdon, England) and cut using a Vibratome UltraPro 5000 cryostat (American Instrument, Haverhill, MA).

### Immunocytochemistry

Immunostaining was performed using the previously established protocol in our lab [19]. Frozen 25 μm coronal sections encompassing the entire tumor were generated from each mouse brain. The sections were blocked with 5% normal goat serum/5% normal horse serum (Vector lab., Burlingame, CA) in PBS containing 0.3% Triton X-100 and 0.05% Phenylhydrazine for 30 minutes and then incubated with monoclonal mouse anti-Pyk2 antibody, dilution 1:1000 (Cell Signaling; #3480S), polyclonal rabbit anti-Iba1 antibody (Wako; #019–19741) 1 μg/ml, and monoclonal GFAP-Cy3 antibody produced in mouse (Sigma, #C9205), dilution 1:500, in PBS-TAT (0.3% TritonX-100, 5% normal goat/5% normal horse serum, 1% sodium azide, 0.01% thimerosal) overnight at 4°C. The sections were incubated with corresponding secondary antibodies (AMCA anti-mouse IgG and fluorescein anti-rabbit IgG (Vector Lab., Burlingame, CA)) overnight and visualized using an Olympus Fluoview FV1000 confocal microscope with 10× objectives or 40× oil immersion objectives.

### Western Blot Analysis

Glioma cells were seeded (1X10^5^) in 60mm dishes and incubated in normal conditions 24 hours prior experiments. The cells were then treated for 2 hours with medium conditioned from glioma/microglia co-culturing (AMCM). Control glioma cells were treated with medium conditioned from microglia or glioma cells alone (MCM or GCM respectively). After 2 hours cells were lysed and clarified cell lysates were separated on 10% SDS-PAGE gels. The proteins were transferred to a PVDF membrane and probed with rabbit polyclonal anti-phospho-Pyk2(Tyr 579/580) primary antibody (Invitrogen; #44636G), dilution 1:1000, followed by anti-rabbit conjugated immunoglobulins (Sigma). Final detection was performed with enhanced chemiluminescence methodology (SuperSignal West Dura Extended Duration Substrate; Pierce, Rockford, IL) and the intensity of the signal was measured using a gel documentation system (Versa Doc Model 1000, Bio Rad). In all cases, intensity of the chemiluminescence signal was corrected for minor changes in protein content after densitometry analysis of the India ink stained membrane.

### Model of local microglial ablation

A model of local microglial ablation using CD11b-HSVTK transgenic mice expressing the herpes simplex thymidine kinase (HSVTK) protein in microglia and macrophages [9, 36] was employed. When these animals are exposed to ganciclovir (GCV), the cells that express HSVTK are eliminated. CD11b-HSVTK (+/-) male mice (gift of Dr. Tsirka, Stonybrook University) were bred with C57BL/6 males. Offspring were genotyped by PCR using primers 5′ -GACTTCCGTGGCTTCTTGCTGC-3′ and 5′ -GTGCTGGCATTACAGGCGTGAG-3′. GL261 glioma cells were implanted into the right cerebral hemisphere of 12–16 week old CD11b-HSVTK (+/-) mice. C57BL/6 mice were used as controls. Tumors were allowed to grow for ten days and then mini-osmotic pumps (Alzet, DURECT, model 2004) were installed for local administration of GCV (Calbiochem, Billerica, MA, USA) to the tumor. Animals were anesthetized and a 3mm brain infusion cannula connected to pump was set up at the previous tumor implantation site using brain infusion kit (Alzet, DURECT). The pumps were placed subcutaneously on the mouse back. The drug was infused at 0.25 μL/h, 1 mg/mL over 7 days. Pumps with normal saline solution were used as a control.

### Percoll purification of glioma cells from tumor tissue

Tumors were removed from the mouse brains, dissected to 1–2 mm pieces with a razor blade, and homogenized using a non-enzymatic cell dissociation solution (Sigma-Aldrich, St. Louis, MO, USA). Glioma cells were purified from the homogenized tissue using Percoll (Sigma-Aldrich, St. Louis, MO, USA) gradients of 30%, 37% and 70%. Following this procedure the glioma fraction was collected from the top and the distinct white ring of microglia cells was collected at the interphase of 37% and 70% Percoll levels. Both glioma and microglia fractions were used for further analysis. Purification efficiency was tested by western blot analysis using mouse monoclonal primary antibodies that detect Iba1, in dilution 1:200 (#1022–5 Lot: GR40934-12 Abcam, Cambridge, MA, USA).

### PF-562,271 inhibition of Pyk2

PF-562,271 (MedKoo Biosciences, #202228) is a potent, ATP-competitive, reversible inhibitor of FAK and Pyk2 catalytic activity with IC(50) of 1.5 and 14 nmol/L, respectively [37]. In the present study, it was used at concentrations 5nM and 16nM to block FAK alone and FAK and Pyk2 together in glioma cells. A 1mM PF-562,271 stock solution was prepared by dilution in water.

### RNA Interference by Small Double-stranded RNAs

Glioma cells were transfected with siRNA targeting Pyk2 (Qiagen, cat. # SI02225321) using HiPerfect Transfection reagent according to the manufacturer’s instructions (Qiagen) and as we previously described [38]. Briefly, 100μL of serum free medium containing 2μL of 20 nM Pyk2 siRNA and 20μL of HiPerfect were prepared and incubated for 30 minutes at room temperature. This complex was then added to 5.0X10^4^ glioma cells containing 1.9mL of cell culture medium in a drop-wise fashion and the plate gently swirled to evenly distribute the transfection complex. In addition, mock transfections were performed and used as control where 100μL of serum free medium containing 20μL of HiPerfect without siRNA was added to the cells. Based on preliminary time course experiments (data not shown), a time point of 3 days after transfection was used. Efficiency of Pyk2 knock-down was determined using western blot.

### Statistical Analysis

Results are expressed as mean ± standard deviation (SD). Statistical probability was calculated using GraphPad software. Unpaired t-tests or one-way ANOVA tests followed by the Tukey’s post-hoc test were used to determine significance between groups. P-values of less than 0.05 were considered as significant.

## Results

### Factors released from microglia stimulate migration and invasion of glioma cells

**Fig 1:**
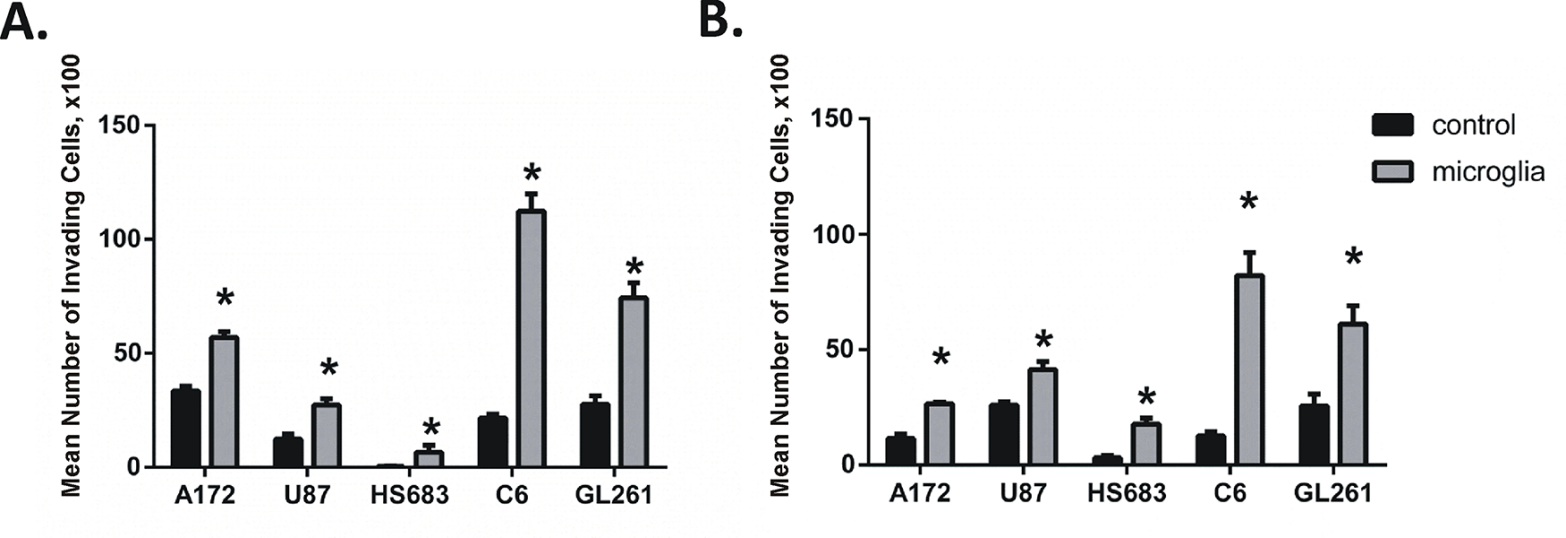
Factors released from microglia increase glioma invasiveness and migration. Invasion **(A)** and migration **(B)** assays were performed. Number of invading and migrating glioma cells without (control) and with microglia in the lower compartment. Results are presented as mean ± S.D. with significant differences from controls (*) shown (p < 0.05). Unpaired t-tests were used to determine significance between groups.

In order to separate the microglial effects on extracellular matrix degradation and on increasing the migratory ability of glioma cells, we performed migration and invasion assays for glioma cells. The data revealed that in the presence of microglia both glioma cell migration and invasion were significantly increased for all investigated glioma cell lines (Fig 1), including HS683 low graded glioma that has low basic level of invasiveness (Fig 1A). The data in the literature indicate that microglia residing in the tumor promote glioma invasion through release of a wide specter of proteases and degradation of the extracellular matrix and this is supported by our finding that microglia increase glioma cell invasion through Matrigel. Furthermore, our data demonstrate that microglia stimulate glioma cell migration without the presence of Matrigel suggesting that microglia promote glioma invasiveness not just by degrading the extracellular matrix, but also by direct activation of glioma cell mobility.

### Factors released from microglia activate the Pyk2 pathway in glioma cells

We used a Panorama Antibody Microarray to survey potential intracellular signaling pathways that might be involved in microglial activated glioma cell migration. This microarray has 112 antibodies spotted and allows the relative levels of key proteins to be measured with a special emphasis on cell signaling proteins known to be involved with apoptosis, cell cycle, cytoskeleton, signal transduction and nuclear proteins. Our data indicate that 24 hours exposure of C6 glioma cells to microglia conditioned medium (MCM) leads to alteration of the expression levels of multiple signaling proteins in C6 cells. For further investigation we selected signaling proteins known to be involved in regulation of invasion and migration. Data are presented in Table 1. We determined that soluble factors released from microglia and contained in MCM up-regulate the expression of Epidermal Growth Factor Receptor (EGFR), Phospholipase C gamma 1 (PLCγ1) proteins, and most markedly, it significantly increased the phosphorylation levels of Pyk2 at Tyr 579/580 (Table 1), thereby stimulating the Pyk2 signaling pathway in glioma cells. All these data indicate that Pyk2 could carry out the signaling initiated by soluble factors released by microglia in order to regulate glioma cell migration. To extend and confirm our finding, we evaluated the localization and activity of Pyk2 in human and rodent glioma cell lines using immunocytochemical and western blot analysis.

**Table 1:** List of selected proteins that are altered in C6 glioma cells after treatment with microglia conditioned medium determined using Panorama Antibody Array System.

Protein	Control/MCM,mean ± SEM	N	P value	P<0.05
EGFR	2.333±0.400	4	0.0072	yes
PLA2	1.855±0.160	4	0.0004	yes
PLCγ1	7.700±2.954	3	0.0209	yes
Pyk2	1.370±0.151	6	0.0543	no
pPyk2 (579/580)	4.932±1.663	5	0.0457	yes

Only proteins that belong to signaling pathways related to migration were selected. The level of proteins was measured as a relative density of fluorescent signal. N indicates the number of repeated experiments.

**Fig 2:**
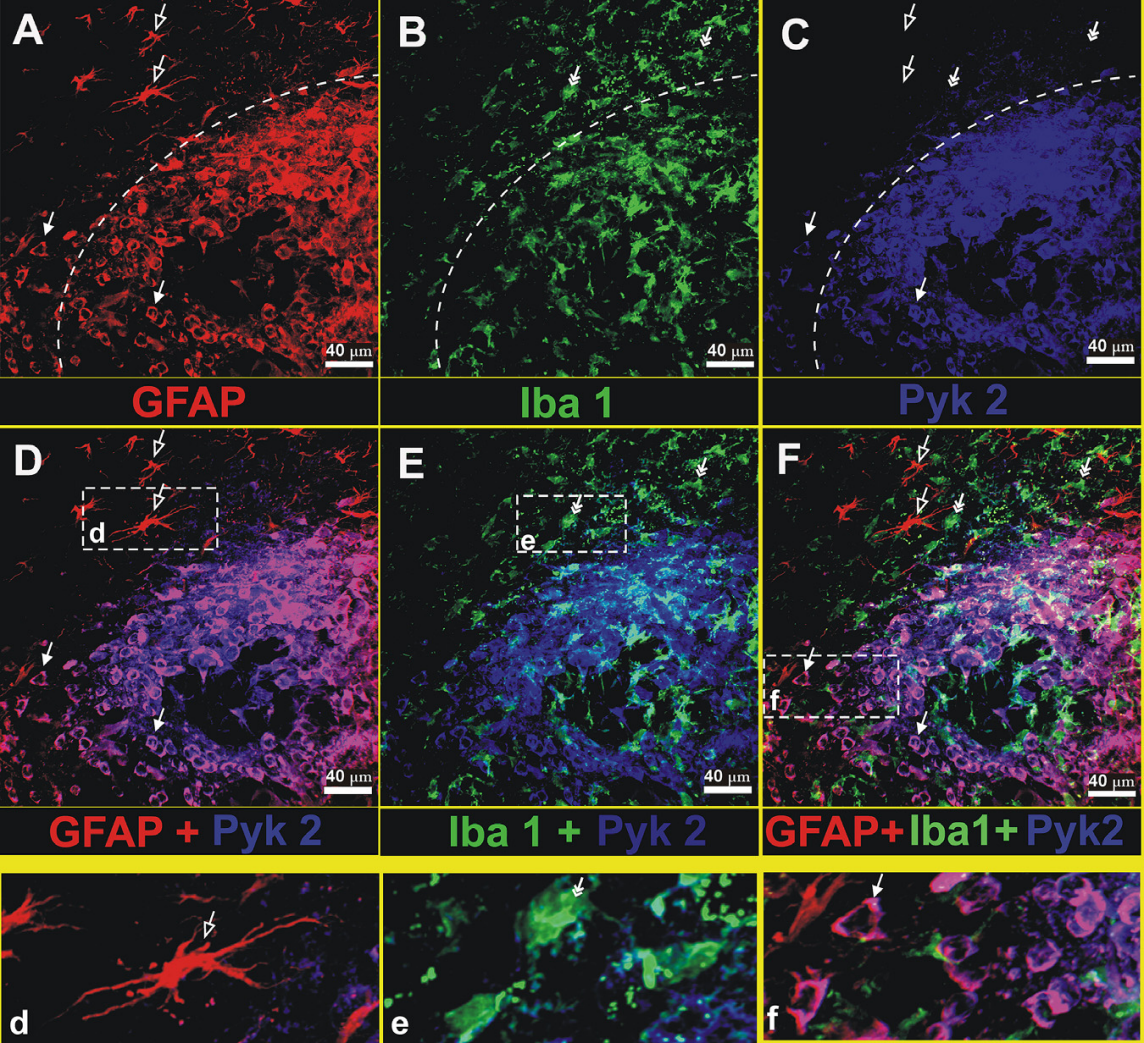
Pyk2 is mostly detected in glioma cells rather than in other cell types in mouse brain. Immunohistochemistry was performed on C57BL/6 mice brain sections containing the tumor area. GL261 glioma cells were implanted into the brains of C57BL/6 mice and grown for 16 days. Photographs show the tumor and surrounding healthy tissue. The dash line outlines the border of tumor. Anti-GFAP antibody was used to detect glioma cells and astrocytes (red, panel A), anti-Iba 1 antibody was used to detect microglial cells (green, panel B) and Pyk2 detection is presented in blue (panel C). The merged images of anti-GFAP and anti-Pyk2, of anti-Iba1 and anti-Pyk2, and of all antibodies together can be seen in merged image boxes D, E, F correspondingly. Insert panels **d**, **e**, and **f** represent enlarged images of astrocytes, microglia, and invading glioma cells. Solid arrows indicate glioma cells, frame arrows indicate astrocytes, and double headed arrows indicate microglia. Scale bar: 40 μm.

To detect the cellular localization of Pyk2 within a brain tumor, we utilized a murine glioma model. Fig 2 shows immunocytochemical analysis of Pyk2, glial fibrillary acidic protein (GFAP, a marker of glioma cells and astrocytes) and Iba1 (a marker of microglial cells) in brain slices obtained from tumor bearing C57BL/6 mice. Brain sections were prepared 16 days after the implantation procedure. Since glioma cells are much larger in size and do not have long branched processes characteristic for astrocytes, glioma cells and astrocytes can be easily distinguished from each other (Fig 2A, 2d and 2f), as we described earlier [35]. The merged image demonstrates that glioma cells express abundant levels of Pyk2 (Fig 2D and 2F) in the tumor core as well as at the invasion area (Fig 2f), whereas astrocytes and microglia do not show a marked amount of Pyk2 (Fig 2D, 2E and 2F). Although there have been previous reports indicating that Pyk2 is present in microglia [39, 40], it is weakly detected in microglia in the present study because the imaging settings were optimized to visualize the large quantities of Pyk2 present in glioma cells.

**Fig 3:**
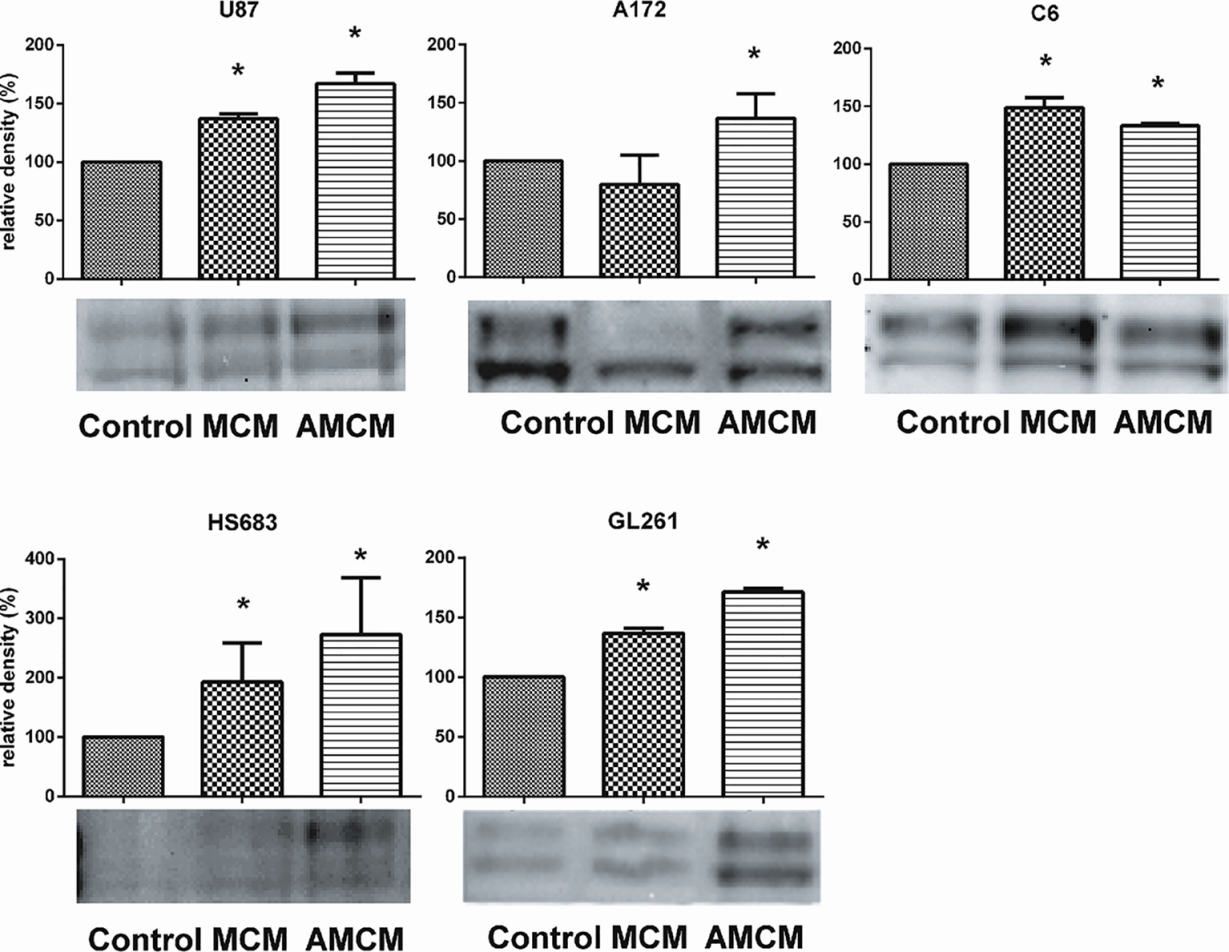
Factors released from microglia upregulate phosphorylation of Pyk2 in glioma cells. Western blot analysis of pPyk2 (Tyr 579/580) protein in glioma cell lines. The signal is detected at the area corresponding to molecular weight of 116 kDa. The graph shows the density of protein in MCM and AMCM treatments relative to control. Two bands in pPyk2 (Tyr 579/580) detection identify phosphorylation in one or both sites. Intensity of the chemiluminescence signal was corrected for minor changes in protein content after densitometry analysis of the India ink stained membrane. The India Ink stained membranes are provided in S1 Fig. The “relative density” axis for HS683 cells is shown in a higher grid scale compare to other cell lines due to higher relative up regulation of Pyk2 phosphorylation in this cell line. Results are presented as mean ± S.D. with significant differences from control (*) (p < 0.05). One-way ANOVA followed by the Tukey’s multiple comparison test was used to determine significance between MCM or AMCM groups compared to control. 5 repeated experiments (N = 5) for each cell line were used for statistical analysis.

To determine if Pyk2 signaling in glioma cells is activated by soluble factors released from microglia, we detected the levels of Pyk2 phosphorylated at Tyr579/580 using western blot. Fig 3 demonstrates the relative levels of phosphorylated Pyk2 in control glioma cells, glioma cells treated with medium conditioned from microglia (MCM) or medium conditioned from microglia that was first co-cultured with glioma (AMCM) to simulate the cross-talk that generally occurs in the tumor microenvironment. Microglia change the pattern of cytokines and other soluble factors released when they are in close vicinity with glioma cells [7, 8, 15, 16]. For this reason, we investigated the effect of soluble factors released from microglia that never received any signaling from glioma cells (MCM) and the effect of soluble factors released by microglia previously interacting with glioma cells (AMCM). Two hours treatment with conditioned mediums were used since we found this treatment duration as an optimum in order to obtain full value effect on Pyk2 phosphorylation in glioma cells. The longer treatment provided steady effect without any future dynamic. The data indicate that all investigated glioma cell lines represent different levels of basic Pyk2 phosphorylation (Fig 3, S1 Fig) with the A172 demonstrating the highest and the HS682 the lowest pPyk2 phosphorylation (the pPyk2579/580 was almost undetected by western blot analysis in untreated HS683 compare to other cell lines). Significant up regulation of Pyk2 phosphorylation was detected in C6, HS683, U87 and GL261 glioma cells 2 hours after treatment with MCM. But even more prominent effect on Pyk2 phosphorylation was found out in all investigated cell lines after treatment with AMCM, with the strongest effect in glioma cells with the low basic level of Pyk2 phosphorylation: U87 and HS683 (70% and 150% up regulation correspondingly), compare to glioma cells with high basic level of Pyk2 phosphorylation: A172 and C6 (40% up regulation). The stronger effect of AMCM compared to MCM suggests that microglial cells change the composition of released soluble factors when they interact with glioma cells and this leads to additional activation of Pyk2 signaling in glioma cells.

In order to exclude possible paracrine effects of glioma cells in response to microglial soluble factors, an additional treatment with glioma conditioned medium (GCM) was used for glioma cells. For this purpose glioma cells were treated with medium, conditioned from other glioma cells pretreated with AMCM. Western blotting did not reveal any quantitative difference in Pyk2 phosphorylation in control and GCM treated cells, indicating that paracrine stimulation does not make significant contribution to microglial activation of Pyk2 in glioma cells.

**Fig 4:**
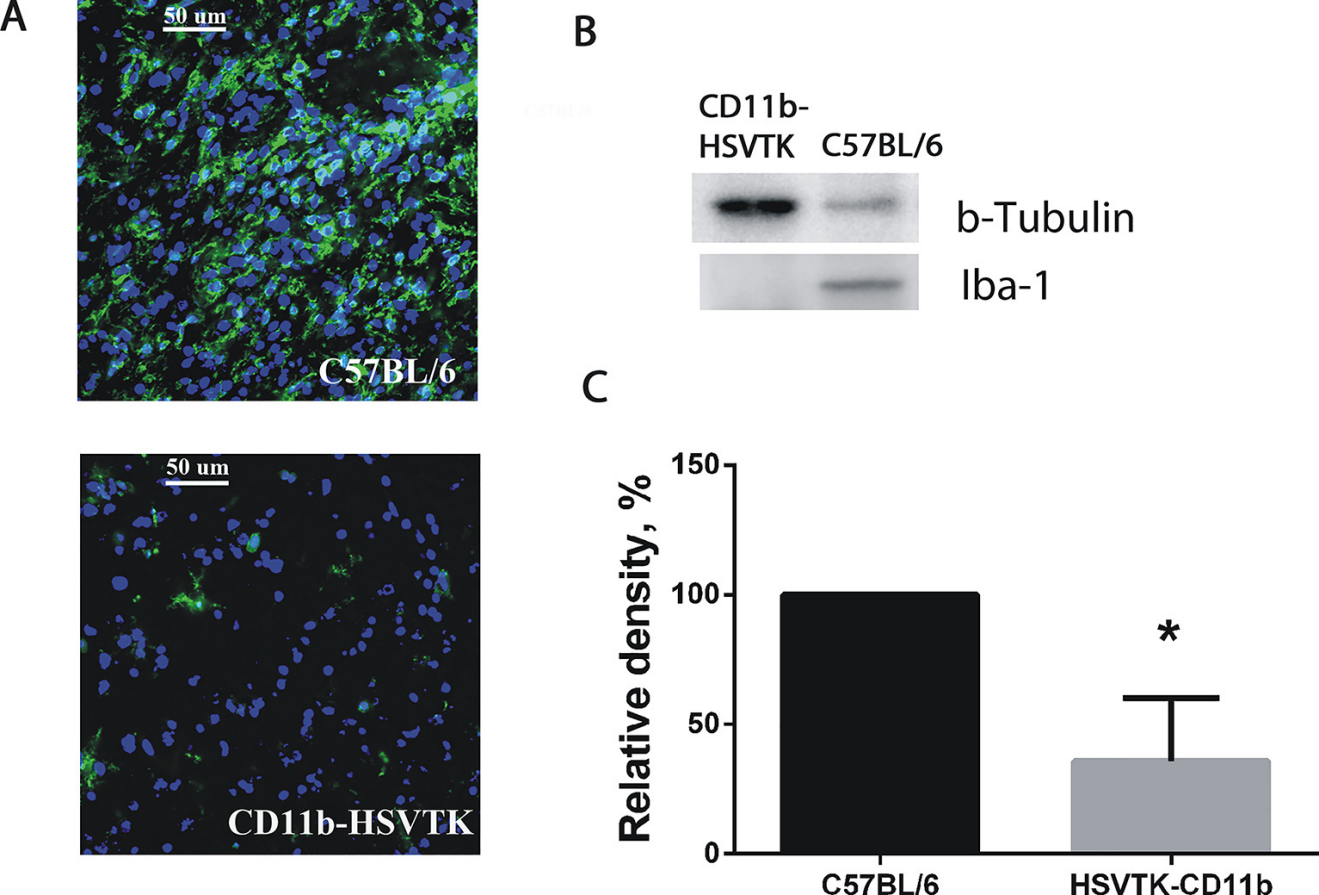
Microglia ablation in brain tumors using the CD11b-HSVTK/GCV system. **(A)** Immunocytochemical detection of microglia in tumors developed in C57BL/6 and CD11b-HSVTK mice brains after local GCV administration. The tumors were generated by intracranial implantation of GL261 glioma cells. GCV was delivered to the tumor area through mini-osmotic pumps. Image shows the significant reduction of microglia in tumors developed in CD11b-HSVTK compared to C57BL/6 mice. Anti-Iba 1 antibody was used to detect microglial cells (green) and DAPI was used to detect all cell nuclei (blue). **(B)** Western blot detection of Iba-1 in tumors extracted from C57BL/6 and CD11b-HSVTK mice brains after the treatment with GCV. (C) The graph shows corresponding levels of Iba-1 protein expression in C57BL/6 and CD11b-HSVTK mice brain tumors after GCV administration determined by western blot. Mean ± S.E and significant difference from control (*) are shown (p < 0.05).

To further evaluate the participation of microglia in activation of the Pyk2 pathway in glioma cells we used a murine model of local microglia ablation. Our preliminary investigation with use of western blot and immunohistochemical staining did not reveal any difference in microglial infiltration of implanted tumors in CD11b-HSVTK and C57BL/6mice without administration of GCV. In order to assess the effectiveness of microglia ablation in tumor site, animals were sacrificed after 7 days of GCV administration and frozen brain sections were prepared followed by immunocytochemical detection of Iba-1. The images of tumors presented in Fig 4A demonstrate that microglial cells were significantly reduced in CD11b-HSVTK mice compared to C57BL/6 animals where microglial cells were still present. For further evaluation of the efficacy of microglia ablation in CD11b-HSVTK mice, quantitative analysis of Iba-1 by western blot was performed in C57BL/6 and CD11b-HSVTK mice brain tumor samples (Fig 4B and 4C). Results showed a significant reduction in the expression of Iba-1 in CD11b-HSVTK mice, confirming the substantial reduction of brain microglia in CD11b-HSVTK as compared to wild type animals.

**Fig 5:**
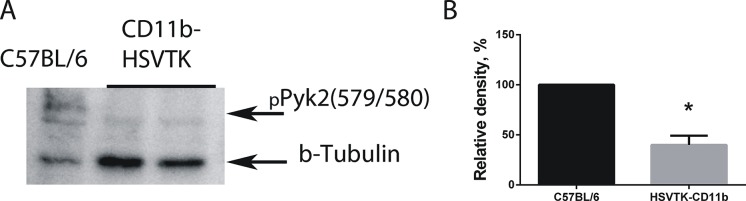
Local microglia ablation in tumor area reduces phosphorylation of Pyk2 in implanted GL261 glioma tumors in CD11b-HSVTK transgenic mice. **(A)** Western blot detection of pPyk2 (Tyr 579/580) protein in glioma cells extracted from C57BL/6 and HSVTK-CD11b mice brains after treatment with GCV. Glioma cells were purified using Percoll gradients. **(B)** The graph shows the quantification of corresponding levels of pPyk2(Tyr 579/580) detected by western blot. Mean ± S.E and significant difference from control (*) is shown (p < 0.05).

In order to evaluate if Pyk2 phosphorylation in glioma cells depends on the grade of microglial infiltration of the tumor we performed western blot analysis of Pyk2(Tyr 579/580) in glioma cells extracted from tumors of C57BL/6 and CD11b-HSVTK mice after GCV administration. Taking into account that Pyk2 can also be expressed in other cell types that might be present in tumor, such as microglia [39, 41], we purified glioma cells extracted from tumor tissue using Percoll gradients. The efficacy of glioma cell purification is presented in S2 Fig. Western blotting revealed the significantly low level of Pyk2 phosphorylation in glioma cells in CD11b-HSVTK mice compared to C57BL/6 after GCV administration (Fig 5). These results match closely with our in vitro data indicating that microglia is involved in the regulation of the Pyk2 signaling in glioma cells.

### Pyk2 pathway is involved in microglial activated glioma cell migration

**Fig 6:**
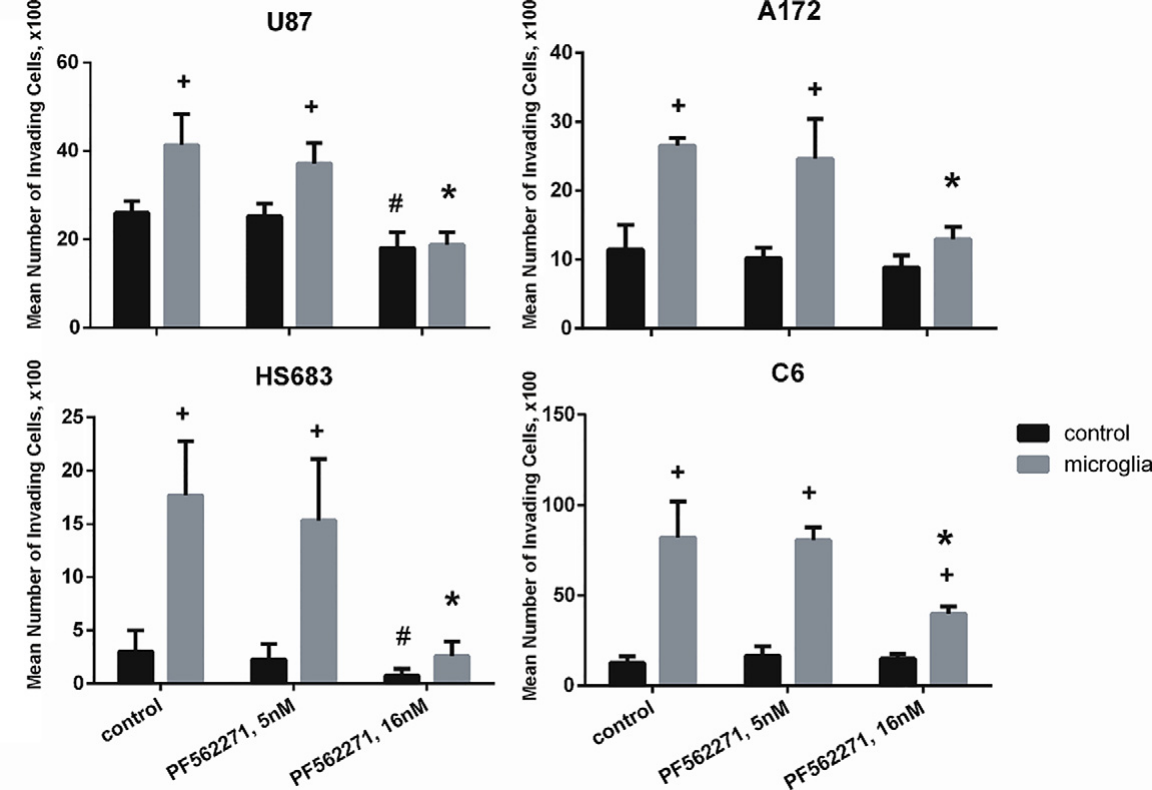
FAK is not involved in microglial stimulation of migration in glioma cells. Data obtained from migration assays for glioma cells. Cells were treated with 5nM (concentration that effectively blocks FAK) and 16nM (concentration that effectively blocks Pyk2) of PF-562,271. Due to different migration abilities in all presented cell lines the Y-axis scales are adjusted for each cell line in order to demonstrate the absolute numbers of migrating cells. Results are presented as mean ± S.D. with significant differences from control in each group (+), from Mock without microglia on the bottom (#), or from Mock with microglia on the bottom (*) (p < 0.05). One-way ANOVA followed by the Tukey’s multiple comparison test was used to determine significance between groups.

Our data indicate that microglia stimulate migration of glioma cells and that treatment of glioma cells with AMCM increases their levels of phosphorylated Pyk2. To assess the involvement of microglia on activation of Pyk2 signaling in glioma cells and on glioma cell mobility, we performed migration assays for glioma cells in the presence and absence of the Pyk2/FAK inhibitor, PF-562,271. PF-562,271 readily passes the blood-brain barrier and selectively blocks FAK at 5 nM and blocks both FAK and Pyk2 at 16 nM [37], S3 and S4 Figs Therefore, we investigated both FAK and FAK/Pyk2 selective doses of PF-562,271. PF-562,271 was given two hours prior to and during the assay. The lower (FAK selective) concentration of PF-562,271 (5 nM) had no effect on glioma cell migration whether microglia were present or not (Fig 6) indicating that FAK was not involved in glioma cell migration. The higher concentration of PF-562,271 (16 nM) effectively eliminated the effect of microglia on glioma cell migration in all investigated glioma cell lines and also reduced the basal migration rates of U87 and HS683 glioma cells in the absence of microglia (Fig 6). Pyk 2 blockade (16 nM PF-562,271) did not alter the basal migration rates of A172 and C6 cells, but significantly reduced migration of these cells when stimulated by microglia (Fig 6).

**Fig 7:**
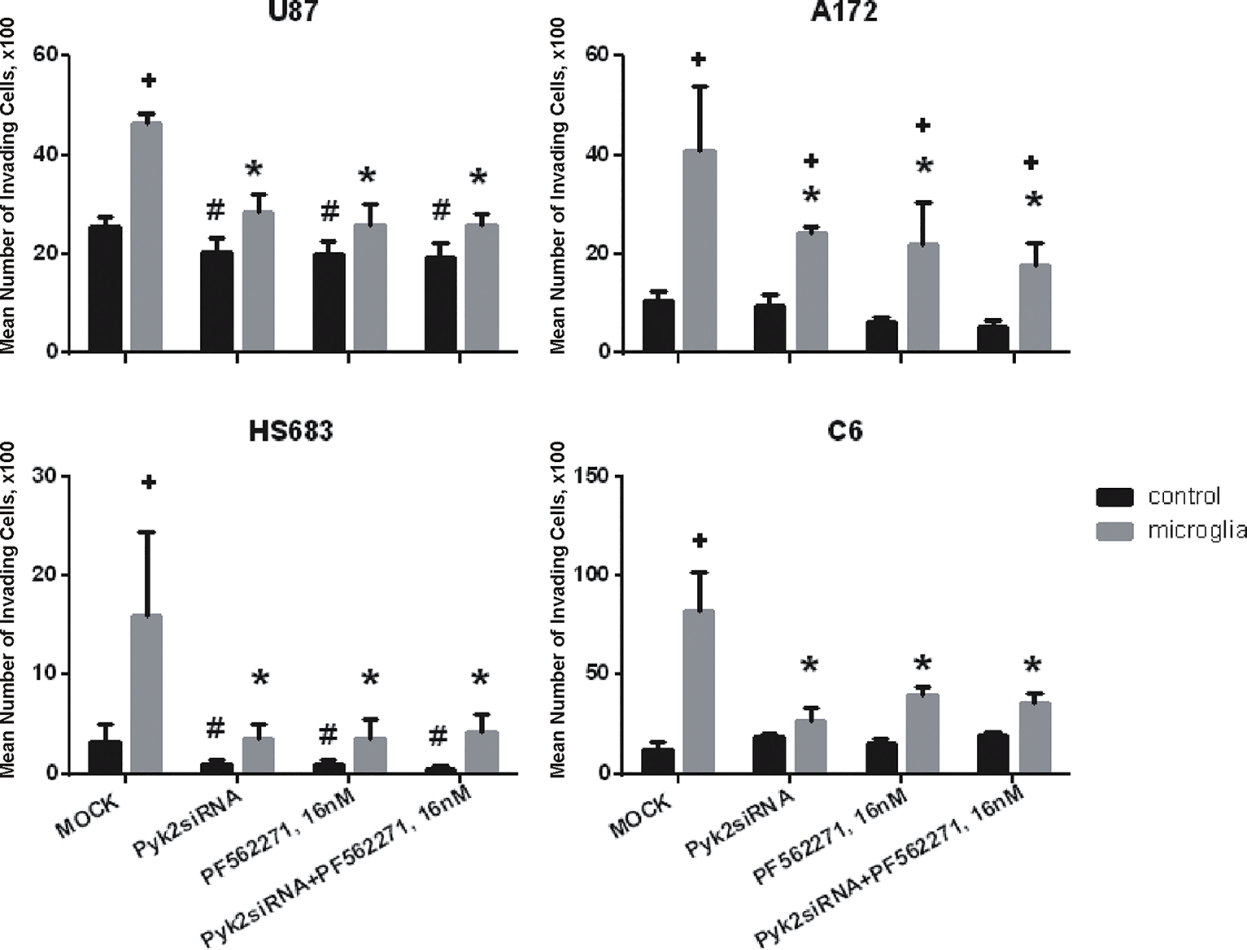
siRNA knock-down and/or pharmacological blockade of Pyk2 by PF-562,271 eliminates the stimulatory effect of microglia on glioma cell migration. Data obtained from standard migration assays for control glioma cells and cells transfected with siRNA against Pyk2 with and without additional application of PF-562,271 in the presence and absence of microglia in the lower compartment. Due to different migration abilities in all presented cell lines the Y-axis scales are adjusted for each cell line in order to demonstrate the absolute numbers of migrating cells. Results are presented as mean ± S.D. with significant difference from control in each group (+), from Mock without microglia on the bottom (#), or from Mock with microglia on the bottom (*) (p < 0.05). One-way ANOVA followed by the Tukey’s multiple comparison test was used to determine significance between groups.

As an independent measure of microglial activation of Pyk2 signaling in glioma cells and its role in glioma cell migration, we used siRNA targeting Pyk2 to selectively knock-down Pyk2 in glioma cells. Three days after transfection with siRNA, levels of Pyk2 were reduced by 60% in glioma cells (S5 Fig). The results obtained using siRNA knock-down of Pyk2 in glioma cells were identical to those obtained using 16 nM PF-562,271 to block Pyk2 (Fig 7). In addition, simultaneous knock-down of Pyk2 using siRNA together with application of 16nM PF-562,271 did not produce further reduction of migration induced by microglia compared to Pyk2 knock-down alone. Taken together the experiments shown in Figs 6 and 7 indicate that Pyk2, but not FAK, is the major signaling pathway involved in microglial stimulation of glioma cell migration.

## Discussion

A large number of studies have described a supportive role of microglia and brain macrophages in tumors [5, 18, 42, 43, 44]. The natural defense mechanisms provided by microglia do not function properly within glioblastomas. Instead, the tumor cells control microglia to support tumor growth, invasion, angiogenesis, and survival [7, 8, 20, 45, 46, 47]. Growing evidence suggests that microglia and macrophages provide the main source of tumor-promoting molecules, such as matrix metalloproteinases, cathepsins and other endopeptidases that are involved in extracellular matrix degradation and are considered to be the key enzymes involved in glioma invasion [7, 8, 45, 48, 49, 50, 51, 52]. Our results reveal that microglia can promote glioma cell dispersal into surrounding areas through another mechanism that directly stimulates the mobility of glioma cells.

Here we demonstrate that glioma cells expressing a large amount of Pyk2 (A172, C6, GL261) also have strong migration and invasion abilities. Contrary, HS683 that has low basic level of Pyk2 phosphorylation also has low migration and invasion capacity. These data allow making a conclusion that the high basic level of Pyk2 phosphorylation in gliomas correlate with high level of invasiveness.

On the other hand the up regulation of Pyk2 phosphorylation at Tyr 579/580 in response to factors released by microglia was identified in all investigated glioma cell lines. At the same time microglia stimulate invasiveness and migration in glioma cells even in non-invasive HS683 low graded glioma. The pharmacological blockade with PF-562,271 and siRNA knockdown of Pyk2 eliminate the simulative effect of microglia on glioma cell migration and indicate that in the presence of microglia, Pyk2 participates as a regulator of migration stimulated by microglia in all investigated glioma cell lines.

In the tumor microenvironment, glioma cells are found in close interaction with tumor infiltrating microglia and macrophages (Fig 2), and consequently provide the stimulus to permanently activate the Pyk2 pathway and subsequently, glioma cell migration (Fig 1). This conclusion is supported by our data with use of the HSVTK/GCV system that allows us to model a tumor microenvironment deficient in microglia cells in vivo (Fig 5). The Pyk2 pathway is poorly activated in microglia ablated tumors and may explain the less invasive nature of the tumor in these animals, demonstrated in earlier reports [9]. We propose that activation of the Pyk2 pathway in gliomas as a result of glioma-microglial interactions allows aggressive dispersal and invasion of glioma cells into surrounding brain tissues. Additional investigation of Pyk2 expression and phosphorylation levels in different gliomas in correlation with the grade of microglial infiltration and tumor invasiveness are necessary.

Moreover, the contribution of Pyk2 in the regulation of glioma cell migration may vary in different glioma cell lines. Pyk2 blockade reduce the basal migration rates of U87 and HS683 glioma cells, but not A172, GL261, and C6 cells in the absence of microglia. These data indicate that Pyk2 has a key role in the basic migratory activity of U87 and HS683, but not A172, GL261, and C6 cells. These might suggest that in addition to Pyk2 other pathways may be involved in the regulation of basal rates of migration in gliomas such as Insulin-like growth factor binding protein 3 (IGFBP-3) that has been implicated in the pathogenesis of gliomas and was shown to be involved in proliferation and the invasive capacity of glioma cells [53]. Other studies demonstrated that Culllin1 (Cul1) is increased significantly in malignant brain tumors, and that silencing of Cul1 in glioma cells inhibited the cell migration and invasion abilities as well as down-regulated MMP-2 and MMP-9 expression that also greatly contribute to the reduced cell invasion and migration abilities [54]. Golgi phosphoprotein 3 (GOLPH3) is also found to be upregulated in gliomas and involved in glioma cell migration and invasion via the mammalian target of rapamycin (mTOR)-Y-box binding protein-1 (YB1) pathway [55]. Furthermore, in has been shown recently that aquaporins (in particular, AQP1) can be involved in migration of some tumor cells. AQP drives water influx, affect the organization of the cytoskeleton through Lin7/β-catenin, facilitating lamellipodia extension and cell migration [56]. These signaling proteins, in addition to Pyk2, might contribute to the regulation of basal migration rates in different gliomas.

FAK, a close relative of Pyk2 that is involved in migration in different tissues, has also been described to be expressed in gliomas. Reports about FAK participation in glioma cell migration are contradictive. Lipinski [25] demonstrated that FAK does not stimulate glioma cell migration, while other reports indicate that it does [57, 58]. Our data obtained by use of pharmacological blockade of FAK demonstrated that FAK is not involved in migration in all investigated cultured glioma cell lines regardless of the presence of microglia.

Our results using antibody microarrays suggest that factors released from microglia upregulate PLCγ1 and EGFR protein levels in C6 glioma cells. A recent publication demonstrated that microglia stimulate migration of glioma cells through EGFR [59]. Since PLCγ1 controls two Pyk2 activation pathways (elevation of [Ca2+]i and PKC phosphorylation) [60, 61] and PLCγ1 binds to the EGFR resulting in activation of PLCγ1-mediated downstream signaling [62, 63], PLCγ1 and EGFR may be upstream regulators of the Pyk2 pathway. PLCγ1 activation is linked to increased invasion of gliomas [64] making PLCγ1 a likely candidate linking the cell surface receptor (activated by microglia) to the Pyk2 pathway. EGFR is an activator of PLCγ1 [65] and EGFR gene amplification is one of the most frequent alterations occurring in glioblastoma [66] and associated with profuse tumor cell invasion [63, 67, 68, 69, 70]. All these together indicate that PLCγ1 and EGFR might be involved in enhancing migration of glioma cells exposed to microglia and could potentially be acting as upstream regulators of the Pyk2 pathway. Further research must be done in order to build the possible pathway involved in the activation of the Pyk2 protein in glioma cells after their interaction with microglia. It is critical to state that EGFR may not be the only receptor involved in the activation of migration of glioma cells. Other receptors such as AT1 and AT2 Angiotensin II receptors [71] have been associated with poor prognosis in human astrocytomas and are involved in the regulation of genes essential for glioma progression.

## Conclusions

In summary, glioma and microglial cells are involved in a reciprocal interaction in the tumor microenvironment where they modulate the functions and abilities of each other in order to promote tumor progression and invasion. In this interaction microglial cells release soluble factors which activate Pyk2 intracellular signaling in glioma cells and thereby, promote migration of these cells.

## Supporting Information

S1 Fig India ink stained membranes as a loading control for western blot identification of pPyk2(579/580). The figure is given in support of Fig 3 and represents membranes probed with antibodies against pPyk2(579/580) and corresponding India Ink staining used as a loading control for densitometry analysis for A172, U87, HS683, C6, Gl261 glioma cell lines.
(TIF)

S2 Fig Western blot detection of Iba1 microglial marker in total tumor and the glioma fraction purified from total tumor with use of Percoll gradients. GL261 glioma cells were implanted into the brains of C57BL/6 mice. In 16 days the half of tumors were removed and used for glioma cells purification with further western blot analysis. The other half of tumors were preceded directly for western blot analysis without glioma cells purification step. Mouse monoclonal primary antibodies that detect Iba1, in dilution 1:200 (#1022–5 Lot: GR40934-12 Abcam, Cambridge, MA, USA), followed by anti-mouse conjugated immunoglobulins (Cell Signaling) were used.
(TIF)

S3 Fig Western Blot (A) and quantification of pPyk2(579/580) protein levels (B) for control GL261 cells and cells treated with 5nM and 16nM PF-562,271. Rabbit polyclonal anti-phospho-Pyk2(Tyr 579/580) primary antibody (Invitrogen; #44636G) dilution 1:1000, were used, followed by anti-rabbit conjugated immunoglobulins (Sigma).
(TIF)

S4 Fig Western Blot (A) and quantification of pFAK(576/577) protein levels (B) for control GL261 cells and cells treated with 5nM and 16nM PF-562,271. Anti-pFAK(576/577) primary antibody were used (Cell Signaling Technology, #93305), dilution 1:1000, followed by anti-rabbit conjugated immunoglobulins (Sigma).
(TIF)

S5 Fig Western Blot (A) and quantification of Pyk2 protein levels (B) for control (MOCK transfected) U87 glioma cells and cells transfected with 10 nM siRNA against Pyk2. Monoclonal mouse anti-Pyk2 antibody were used (Cell Signaling; #3480S), dilution 1:1000, followed by anti-mouse conjugated immunoglobulins (Cell Signaling).
(TIF)
