# Supplementary figures and images for: Microglia Activate Migration of Glioma Cells through a Pyk2 Intracellular Pathway
Source: PLoS One. 2015 Jun 22;10(6):e0131059. doi: 10.1371/journal.pone.0131059 (PMC4476590; doi:10.1371/journal.pone.0131059)

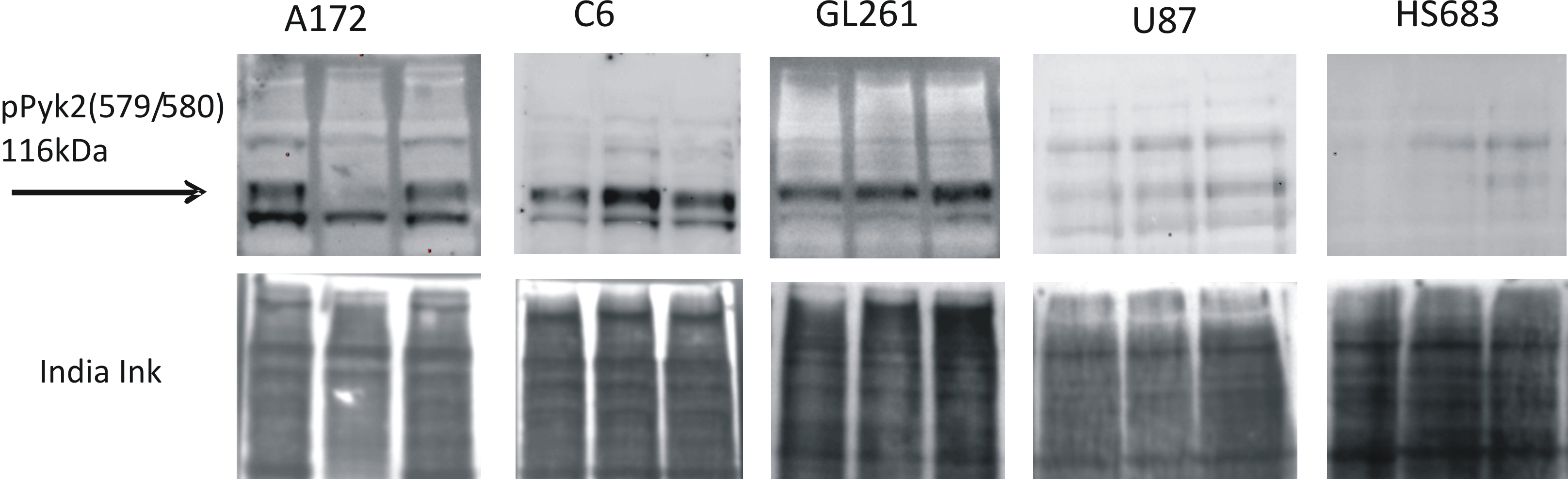

Supplement: S1 Fig — The figure is given in support of Fig 3 and represents membranes probed with antibodies against pPyk2(579/580) and corresponding India Ink staining used as a loading control for densitometry analysis for A172, U87, HS683, C6, Gl261 glioma cell lines. (TIF) [file pone.0131059.s001.tif]

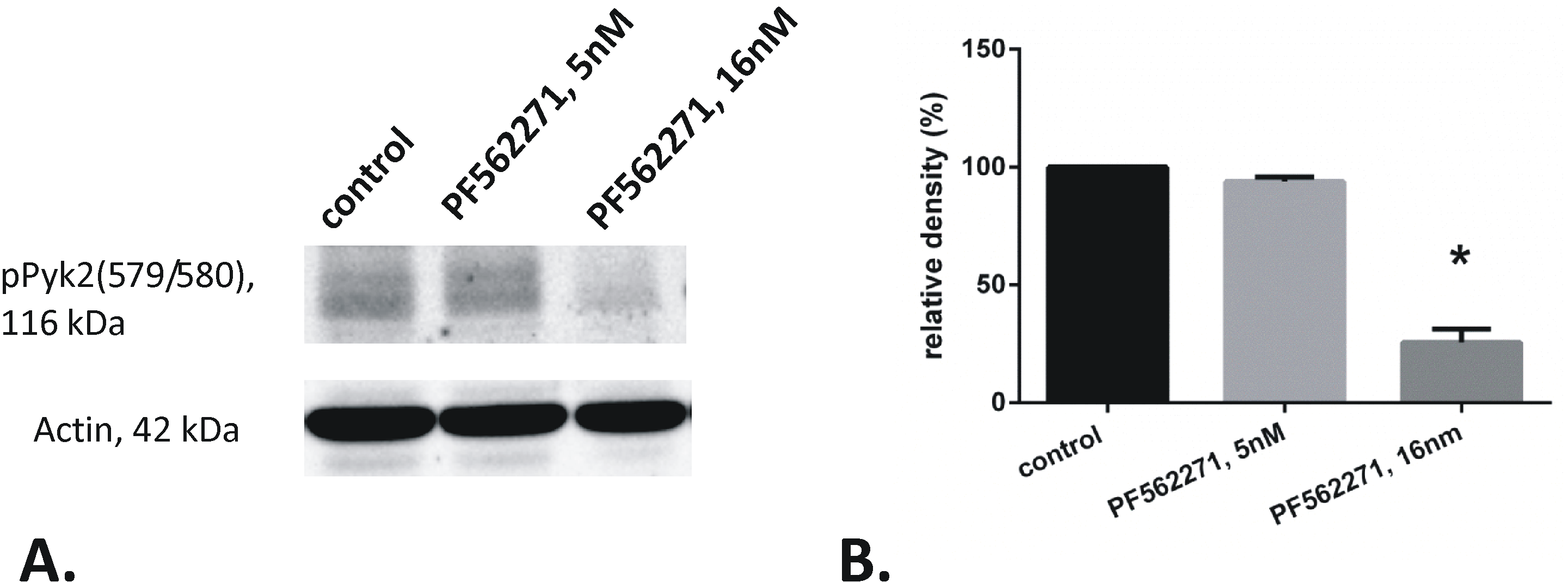

Supplement: S3 Fig — Rabbit polyclonal anti-phospho-Pyk2(Tyr 579/580) primary antibody (Invitrogen; #44636G) dilution 1:1000, were used, followed by anti-rabbit conjugated immunoglobulins (Sigma). (TIF) [file pone.0131059.s003.tif]

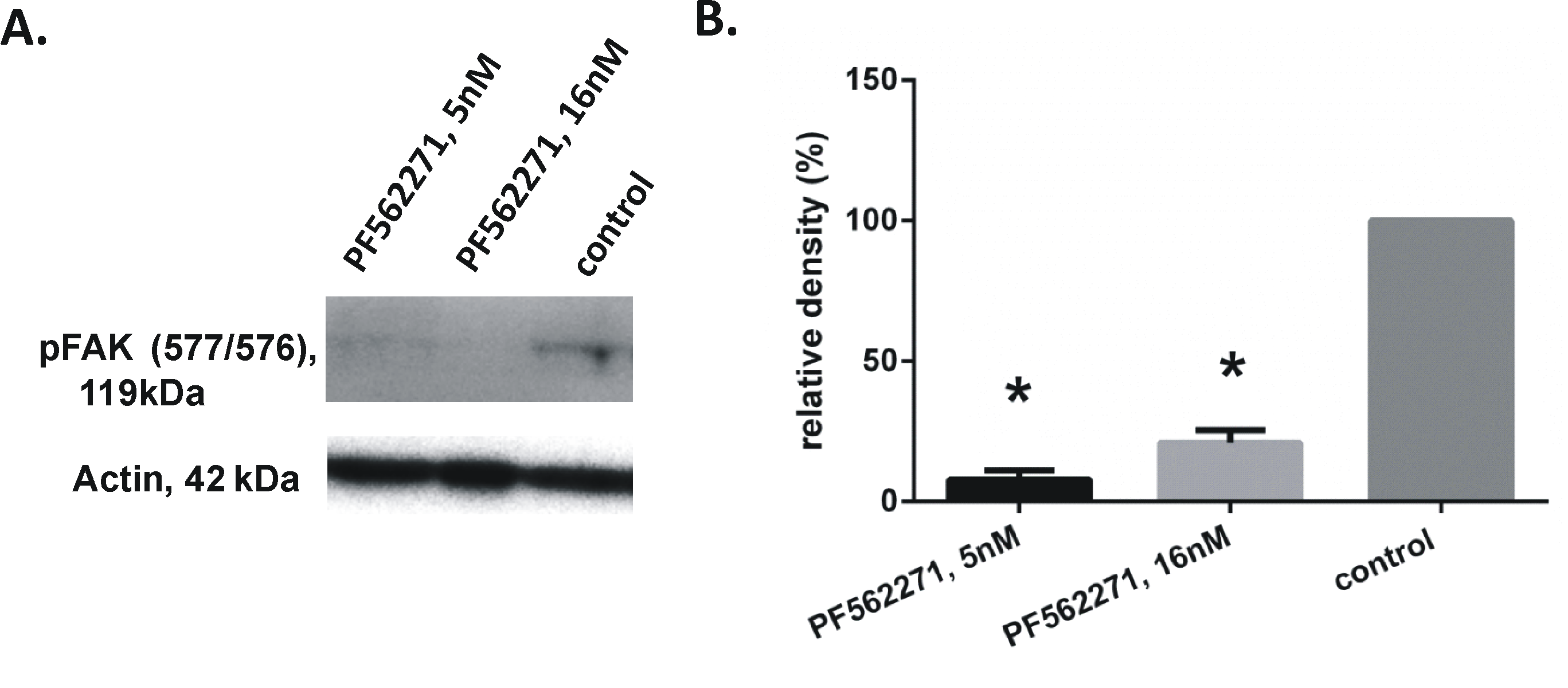

Supplement: S4 Fig — Anti-pFAK(576/577) primary antibody were used (Cell Signaling Technology, #93305), dilution 1:1000, followed by anti-rabbit conjugated immunoglobulins (Sigma). (TIF) [file pone.0131059.s004.tif]

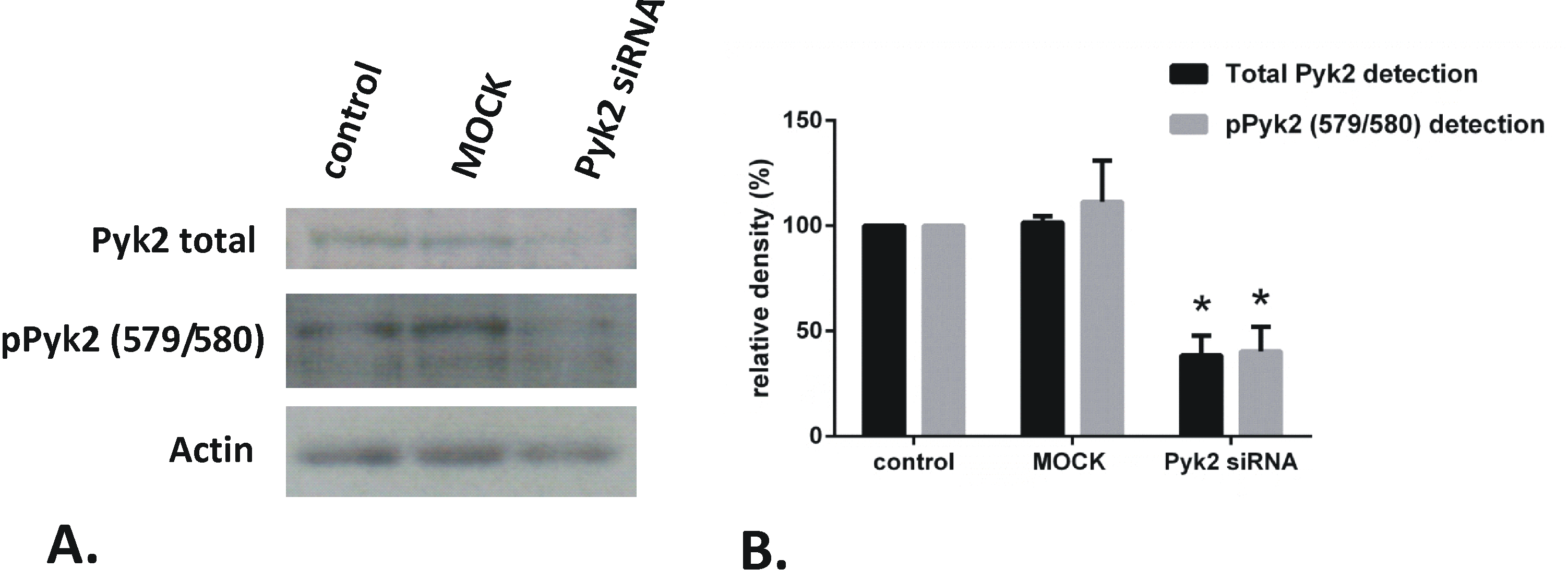

Supplement: S5 Fig — Monoclonal mouse anti-Pyk2 antibody were used (Cell Signaling; #3480S), dilution 1:1000, followed by anti-mouse conjugated immunoglobulins (Cell Signaling). (TIF) [file pone.0131059.s005.tif]
